# The severity of pandemic H1N1 influenza in the United States, April – July 2009

**DOI:** 10.1371/currents.RRN1042

**Published:** 2010-04-19

**Authors:** Anne M Presanis, Marc Lipsitch, Daniela De Angelis, Swine Flu Investigation Team, New York City Department of Health and Mental Hygiene, Angie Hagy, Carrie Reed, Steven Riley, Ben Cooper, Paul Biedrzycki, Lyn Finelli, Jade B

**Affiliations:** ^*^MRC Biostatistics Unit, Cambridge; ^†^Dept of Epidemiology and Center for Communicable Disease Dynamics, Harvard School of Public Health; ^‡^Health Protection Agency, London and MRC Biostatistics Unit, Cambridge; ^§^City of Milwaukee Health Department; ^¶^Epidemiologist, Centers for Disease Control and Prevention; ^#^The University of Hong Kong; ^**^Health Protection Agency and ^‡‡^CDC

## Abstract

Background

Accurate measures of the severity of pandemic influenza A/H1N1 (pH1N1) are needed to assess the likely impact of an anticipated resurgence in the autumn in the Northern Hemisphere. Severity has been difficult to measure because jurisdictions with large numbers of deaths and other severe outcomes have had too many cases to assess the total number with confidence. Also, detection of severe cases may be more likely.

Methods and Findings

We used complementary data from two US cities: Milwaukee attempted to identify cases of medically attended infection whether or not they required hospitalization, while New York City focused on the identification of hospitalizations, intensive care admission or mechanical ventilation (hereafter, ICU), and deaths. New York data were used to estimate numerators for ICU and death, and two sources of data: medically attended cases in Milwaukee or self-reported influenza-like illness in New York, were used to estimate ratios of symptomatic cases:hospitalizations. Combining these data with estimates of the fraction detected for each level of severity, we estimated the proportion of symptomatic cases that died (symptomatic case-fatality ratio, sCFR), required ICU (sCIR), and required hospitalization (sCHR), overall and by age category. Evidence, prior information and associated uncertainty were analyzed in a Bayesian evidence synthesis framework. Using medically attended cases and estimates of the proportion of symptomatic cases medically attended, we estimated sCFR of 0.048% (95% credible interval, CI 0.026%-0.096%), sCIR of 0.239% (0.134%-0.458%), and sCHR of 1.44% (0.83%-2.64%). Using self-reported ILI, we obtained estimates approximately 7-9x lower. sCFR and sCIR appear to be highest in persons 18 and older, and lowest in children 5-17. sCHR appears to be lowest in persons 5-17; our data were too sparse to allow us to determine the group in which it was the highest.

Conclusions

These estimates suggest that an autumn-winter pandemic wave of pH1N1 with comparable severity per case could lead to a number of deaths in the range from considerably below that associated with seasonal influenza to slightly higher, but with greatest impact in young children and non-elderly adults. These estimates of impact depend on assumptions about total incidence of infection and would be larger if incidence of symptomatic infection were higher or shifted toward adults, if viral virulence increased, or if suboptimal treatment resulted from stress on the health care system; numbers would decrease if the proportion infected or symptomatic were lower.

## NOTE: The final version of this work is now published in *PLoS Medicine*

## INTRODUCTION 

The present pandemic of pandemic influenza A/H1N1 (pH1N1) has resulted in over 209,000 laboratory-confirmed cases and over 3205 deaths worldwide as of September 11 (http://www.who.int/csr/don/2009_09_11/en/index.html, accessed September 14, 2009), but national and international authorities have acknowledged that these counts are substantial underestimates, reflecting an inability to identify, test, confirm and report many cases, especially mild cases.  Severity of infection may be measured in many ways, the simplest of which is the case-fatality ratio (CFR), the probability that an infection causes death.  Other measures of severity, which are most relevant to the burden a pandemic exerts on a health care system, are the case-hospitalization and case-intensive care ratios (CHR and CIR respectively), the probabilities that an infection leads to hospitalization or intensive care unit (ICU) admission.  In the absence of a widely available and validated serologic test for infection, it is impossible to estimate these quantities directly, and in this report we instead focus on the probabilities of fatality, hospitalization and ICU admission per *symptomatic* case; we denote these ratios sCFR, sCHR, and sCIR respectively.

Although it is difficult to estimate these quantities, estimates of their values and associated uncertainty are important for decision making, planning and response during the progression of this pandemic.  Initially, some national and international pandemic response plans were tied partly to estimates of the CFR, but such plans had to be modified in the early weeks of this pandemic, as it became clear that the CFR could not at that time be reliably estimated [1]. Costly measures to mitigate the pandemic, such as the purchase of medical countermeasures and the use of disruptive social distancing strategies may be acceptable to combat a more severe pandemic but not to slow a milder one.  While past experience [2] and mathematical models [3] [4] [5] suggest that between 40% and 60% of the population will be infected in a pandemic with a reproduction number similar to those seen in previous pandemics, the number of deaths and the burden on the health care system also depend on the age-specific severity of infection, which varies by orders of magnitude between pandemics [6] and even between different waves in the same pandemic [7] .   Reports from the Southern Hemisphere suggest that a relatively low fraction of the population experienced symptomatic pH1N1 infection (7.5% in New Zealand, for example [8] ), though these numbers are considered highly uncertain [8].  On the other hand, primary care utilization for influenza-like illness has been considerably higher than in recent years [8], and anecdotal reports in the Southern Hemisphere have indicated that some intensive care units have been overwhelmed and surgery postponed due to a heavy burden of pH1N1 cases [9] [10] .

The problem of estimating severity of pH1N1 infection is also the problem of estimating how many of the infected individuals in a given population and time period subsequently develop symptoms, are medically attended, hospitalized, admitted to ICU, and die due to the virus.  No large jurisdiction in the world has been able to maintain an accurate count of total pH1N1 cases once the epidemic has grown beyond hundreds of cases, because the effort required to confirm and count such cases grows in proportion to the size of the exponentially-growing epidemic [11] , making it impossible to estimate reliably the frequency of an event (death) that occurs on the order of 1 in 1000 patients or fewer.  As a result, simple comparisons of the number of deaths to the number of cases suffer from underascertainment of cases (making the estimated ratio too large), and underascertainment of deaths due to inability to identify deaths caused by the illness and due to delays from symptom onset to death (making the estimated ratio too small) [1] .  Imperfect ascertainment of both numerator and denominator will lead to biased estimates of the case-fatality ratio.  Estimating the number of persons at these varying levels of severity therefore depends on estimating the proportion of true cases that are recognized and reported by existing surveillance systems.  Similar problems affect estimates of key parameters for other diseases, such as HIV.  In HIV, a solution to this problem – which now forms the basis for the UK’s annual HIV prevalence estimates published by the Health Protection Agency [12] [13] – has been to synthesize evidence from a variety of sources that together provide a clearer picture of incidence, prevalence, and diagnosis probabilities. This synthesis is performed within a Bayesian framework that allows each piece of evidence, with associated uncertainties, to be combined into an estimate of the numbers of greatest interest [14] [15] .

Here we use a similar framework to synthesize evidence from two cities in the United States – New York and Milwaukee – together with estimates of important detection probabilities from epidemiologic investigations carried out by the Centers for Disease Control and Prevention (CDC) and other data from CDC.   We estimate the severity of pH1N1 infection from data from spring-summer, 2009 wave of infections in the United States. The New York City and Milwaukee health departments pursued differing surveillance strategies that provided high-quality data on complementary aspects of pH1N1 severity, with Milwaukee documenting medically attended cases and hospitalizations, and New York documenting hospitalizations, ICU/ventilation use, and fatalities. These are the numerators of the ratios of interest.

The denominator for these ratios is the number of symptomatic pH1N1 cases in a population, which cannot be assessed directly.  We use two different approaches to estimate this quantity.  In the first, we use self-reported rates of seeking medical attention for influenza-like illness from several CDC investigations to estimate the number of symptomatic cases from the number of medically attended cases, which are estimated from data from Milwaukee.  In the second, we use self-reported incidence of influenza-like illness (ILI) in New York City, and making the assumption that these ILI cases represent the true denominator of symptomatic cases, we directly estimate the ratio between hospitalizations, ICU admissions/mechanical ventilation, and deaths (adjusting for ascertainment) in New York.   Each of these two methods provides estimates for the general population, and also for broad age categories 0-4, 5-17, 18-64, and 65+ years.   The result of each approach is a tiered severity estimate of the pandemic.  

## METHODS

*Methods Overview:* The overall goal of this study was to estimate, for each symptomatic pH1N1 case, what was the probability of hospitalization, ICU admission or mechanical ventilation, or death, overall and by age group.  The challenge is that in any population large enough to have significant numbers of the severe outcomes, there was no reliable measure of the number of symptomatic pH1N1 cases.  The problem was approached in two ways.  Approach 1 was to view the severity of infection as a “pyramid” [16] ,  with each successive level representing greater severity; to estimate the ratio of the top level to the base (symptomatic cases), we estimated the ratios of each successive level to the one below it (Fig. 1, left side).  Thus we broke down (for example) the sCFR (Fig. 1, black), i.e. the probability of death per symptomatic case, into components for which data were available – the probability of coming to medical attention given symptomatic infection (CDC survey data); the probability of being hospitalized given medical attention (Milwaukee data); and the probability of dying given hospitalization (New York data, including a correction for those who died of pH1N1 but were not hospitalized).   A second approach was to use the self-reported incidence of influenza-like illness from a telephone survey in New York as the estimate of total pH1N1 symptomatic disease, and the total number of confirmed deaths in New York City as the estimate of the deaths (after accounting for possibly imperfect viral testing sensitivity).  In each case, prior distributions were used to quantify information on the probability that individuals at each level of severity were detected; these prior distributions reflected the limited data available on detection probabilities and associated uncertainty.  

 All of these estimates were combined within a Bayesian evidence synthesis framework.  This framework permits the estimation of probabilities for the quantities of interest (the sCFR, sCIR and sCHR) and associated uncertainty (expressed as credible intervals).  These credible intervals appropriately reflect the combined uncertainties associated with each of the inputs to the estimate – mainly, the true numbers of cases at each level of severity, after accounting for imperfect detection – as well as the uncertainties due to sampling error (chance).

*Study populations:*  Data were obtained from enhanced pandemic surveillance efforts by the City of Milwaukee Health Department and the New York City Department of Health and Mental Hygiene.  Details of testing policies, data acquisition, and analysis are given in Appendix.  All data were analyzed first in aggregate and then by age category.

*Milwaukee data:* Between April 6 and July 16, 2009, Milwaukee had recorded 3278 confirmed cases and 4 deaths due to pH1N1, reflecting sustained efforts to test cases of influenza-like illness and their household contacts from the start of the epidemic in April until mid-July.  On April 27, Milwaukee initiated protocols including recommendations for testing persons with influenza symptoms and travel history to a novel H1N1 area, using a reverse-transcriptase polymerase chain reaction (RT-PCR) test specific for pH1N1.  By May 7, Milwaukee issued testing guidance updated to recommend testing persons with moderate to severe symptoms, except health care workers, for whom testing persons with mild symptoms continued to be recommended.  We used a line list dated July 21, and in a preliminary analysis examined the frequency of hospitalization among cases by “episode date” (the earliest date in their case report).  The proportion of confirmed cases hospitalized was stable around 3% up to May 20, after which it increased markedly to 6-8% in the following weeks.  We judged that this change reflected reduced testing of mild cases and limited our analysis (used to inform the ratio of hospitalizations to medically attended cases) to the 763 cases with an episode date up to or including May 20.  While Milwaukee data were not the main source of estimates of ICU admission or death probabilities, we did employ hospitalized cases up to an episode date of June 14 to contribute to estimates of the ratio of deaths or ICU admissions to hospitalizations, since these should not be affected by failure to test mild cases.       

*New York case data: *New York City maintained a policy from April 26 to July 7, 2009 of testing hospitalized patients with influenza-like illness (ILI) according to various criteria.  These criteria changed up to May 12, from which point they remained as follows: all hospitalized ILI patients received a rapid influenza antigen test.  Those patients who tested positive on rapid test (which is known to have low sensitivity for seasonal influenza [17] and for pH1N1 [18] ), and any patient in the ICU or on a ventilator, regardless of rapid test result, received RT-PCR tests for pH1N1.  We obtained a line list of confirmed or probable hospitalized cases dated July 7, and found in preliminary analysis that all patients in this line list had a date (onset or admission) in their record no later than June 30, 7 days prior to the date of the line list.   Given that >90% of hospitalizations were reported in New York within 7 days, we used this entire line list without accounting for delays in reporting of hospitalizations.  Also, given that 98% of admissions occurred after May 12, we did not attempt to account for changes in testing practices prior to May 12.  This line list included a field indicating whether the patient had been admitted to the ICU or ventilated; patients were not followed up after admission to determine if this status changed.  However, a chart review of 99 hospitalized cases indicated that none had been admitted to the ICU after admission, so no effort was made to account for this limitation.

Separately, we obtained a list of 53 deaths attributed to pH1N1, of whom 44 (83%) had been hospitalized before dying.  All patients with known influenza or unexplained febrile respiratory illness at the time of death had post-mortem samples and/or samples from before they died sent for PCR testing.      

*New York telephone survey data: *To estimate levels of ILI in New York City, DOHMH conducted 1,006 surveys between May 20 and May 27, 2009, and 1,010 between June 15 and June 19. Interviews lasted 5 minutes and were conducted with households in both English and Spanish. The survey used a random-digit dialing (RDD) telephone sampling methodology to obtain data from a random sample of residential households in New York City. A non-random individual from each selected household was interviewed and provided information about all household members. Sampled numbers were dialed between 5 and 15 times to contact and interview a household, or until the sampled number was determined to be non-working.

To account for this design, the data were weighted to the 2007 American Community Survey (ACS); respondents were weighted to householders by borough, age, gender, and race/ethnicity, and the population was weighted by age to the borough of residence.    

The survey’s RDD sampling methodology gave a useful overview of ILI in the community, but it has limitations. The design does not include individual living in households only reachable by cellular telephone but not by a landline telephone number, and also omitted those living in group or institutional housing. Although households were randomly selected, for the sake of efficiency, the interviewed adult was not. Instead, an available adult in the household provided information about all household members and themselves, which may have introduced bias.  The results of the survey are being compiled for publication elsewhere.  Here, we use summaries of these results by age group (see Appendix) as one means to provide denominators of symptomatic cases.

*Data on detection probabilities from CDC investigations:  *Sources of data include two community surveys on influenza-like illness (ILI) and health seeking behavior, and two field investigations conducted during early outbreaks of pH1N1 in the United States.  These sources are described in further detail elsewhere [19], but are summarized here briefly.  In 2007, the Behavioral Risk Factor Surveillance Survey (BRFSS), a random-digit dialed telephone survey, included a module on ILI in 9 states.  This module included questions to assess the incidence of ILI, health-seeking behavior, physician diagnosis of influenza, and treatment of influenza with antiviral medications during the annual 2006-2007 influenza season.  In May 2009, following the emergence of pH1N1, a random-digit dialed telephone survey sampled similarly to the BRFSS was conducted in the same 9 states using only the ILI module from the 2007 BRFSS and limited demographic questions.  In addition, some data was available from field investigations conducted during large outbreaks of pH1N1 in one community in Chicago and a university campus in Delaware.  Investigations of these outbreaks consisted of household interviews in a Chicago neighborhood and an online survey of students and faculty in Delaware.  These data were used to inform detection probabilities.  In addition, these data were used to inform a prior distribution on the ratio between symptomatic and medically attended cases, \begin{equation*}c_{M|S}\end{equation*}: these surveys estimated that between 42% and 58% of symptomatic ILI cases sought medical attention [19]. 

**Figure:**
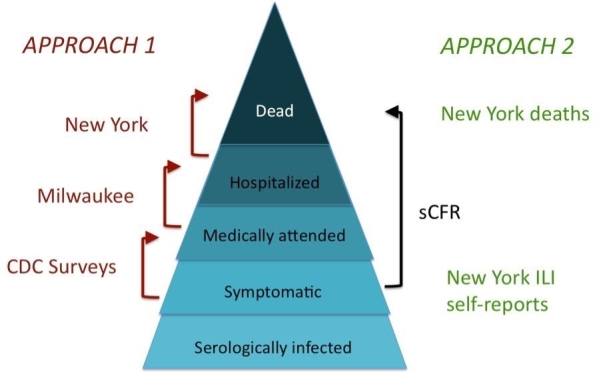
Figure 1.  Diagram of two approaches to estimating the symptomatic case-fatality ratio (sCFR), shown in black.  Approach 1 used three data sets to estimate successive steps of the severity pyramid.  Approach 2 used self-reported influenza-like illness for the denominator, and confirmed deaths for the numerator, both from New York City.  Both approaches used prior distributions, in some cases informed by additional data, to inform the probability of detecting (confirming and reporting) cases at each level of severity (not shown in the diagram; see Appendix).  The Bayesian evidence synthesis framework was used as a formal way to combine information and uncertainty about each level of severity into a single estimate and associated uncertainty that reflected all of the uncertainty in the inputs. 

*Analysis: *Estimation of the probabilities of primary interest, \begin{equation*}c_{H|S}\end{equation*} , \begin{equation*}c_{I|S}\end{equation*} , and \begin{equation*}c_{D|S}\end{equation*}, respectively the sCHR, sCIR, and sCFR,  was undertaken using a Bayesian evidence synthesis framework [14]. Details are given in the Appendix, and a schematic illustration of the model is given in Figure 2.  Briefly, in this framework, prior information about the quantities of interest (including the uncertainty associated with this prior information) is combined with the information coming from the observed cases at each severity level to derive a *posterior distribution* on these quantities. This posterior distribution fully reflects all information about the quantities of interest that is contained in the prior distribution and the observed data. Specifically, it was assumed that detected cases \begin{equation*}O\end{equation*} at each level of severity – medically attended ( \begin{equation*}M\end{equation*} ), hospitalized ( \begin{equation*}H\end{equation*} ), ICU-admitted ( \begin{equation*}I\end{equation*} ), and fatal ( \begin{equation*}D\end{equation*} ) – represented binomially distributed samples from the true number of cases \begin{equation*}N\end{equation*}  at the corresponding level of severity, in the given location (New York, abbreviated \begin{equation*}N\end{equation*}  or Milwaukee, abbreviated \begin{equation*}W\end{equation*} ), with probability equal to the probability of detection at each level ( \begin{equation*}d\end{equation*} *)*.  The probability \begin{equation*}d\end{equation*}  for each level was informed by evidence on the probability of testing at each level of severity (which may have depended on the sensitivity of the rapid test if this was required for PCR testing) and the sensitivity of the PCR test (Table 1).  Thus, for example, we defined the probability of detecting a hospitalized case in New York as \begin{equation*}d_{HN}=d_{HN1}d_{HN2}\end{equation*} , where \begin{equation*}d_{HN1}\end{equation*} was the probability of performing an RT-PCR based test and \begin{equation*}d_{HN2}\end{equation*}  was the sensitivity of that test.  Hence, the observed number of hospitalized patients in New York, \begin{equation*}O_{HN}\end{equation*} , was assumed to be distributed as \begin{equation*}Binomial(N_{HN},d_{HN})\end{equation*} .  

**Figure:**
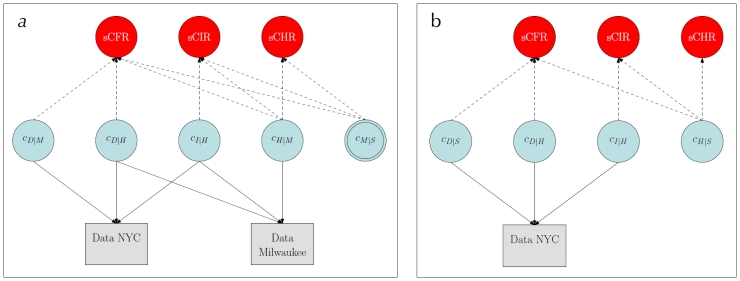
**Figure 2:** Schematic illustration of the relationship between the observed data (rectangles) and the conditional probabilities (blue circles). The key quantities of interest, sCHR, sCIR, and sCFR are products of the relevant conditional probabilities.  (a) Approach 1, synthesizing data from New York City and Milwaukee.   Note that \begin{equation*}c_{M|S}\end{equation*}  (double circle) is informed by prior information [19]rather than observed data.  (b) Approach 2, using data from New York City only, including the telephone survey.  Abbreviations: sCFR: symptomatic case-fatality ratio; sCIR: symptomatic case-ICU (or mechanical ventilation) ratio; sCHR: symptomatic case-hospitalization ratio.  Variables:\begin{equation*}c_{D|M}\end{equation*} : the ratio of deaths to medically-attended cases; \begin{equation*}c_{D|H}\end{equation*}: the ratio of deaths to hospitalized cases; \begin{equation*}c_{I|H}\end{equation*}: the ratio of cases admitted to intensive care or using mechanical ventilation to hospitalized cases; \begin{equation*}c_{H|M}\end{equation*} : the ratio of hospitalized cases to medically attended cases; \begin{equation*}c_{M|S}\end{equation*} : the ratio of medically-attended cases to symptomatic cases;\begin{equation*}c_{D|S}\end{equation*} : the ratio of deaths to symptomatic cases; \begin{equation*}c_{H|S}\end{equation*} : the ratio of hospitalized cases to symptomatic cases.

We noted that the ratios \begin{equation*}c_{H|S}\end{equation*} , \begin{equation*}c_{I|S}\end{equation*} , and \begin{equation*}c_{D|S}\end{equation*} can be built up multiplicatively from simpler components: for instance, the ratio of deaths to symptomatic infections may be expressed as \begin{equation*}c_{D|S}=c_{D|H}c_{H|M}c_{M|S}\end{equation*} , the product of the ratios of deaths:hospitalizations, of hospitalizations:medically attended cases, and of medically attended cases:symptomatic cases.  These ratios of increasing severity are similar to conditional probabilities but are not strictly so in all cases, since for example some deaths in New York City occurred in persons who were not hospitalized.  For this reason we model deaths separately among hospitalized and non-hospitalized patients, i.e. \begin{equation*}c_{D|S}=c_{D|H}c_{H|M}c_{M|S}+c_{D|M}c_{M|S}\end{equation*} .  For each observed level of severity (medically-attended, hospitalized, ICU, death), the true number of cases was modeled as a binomial sample from the true number of cases at an appropriate lower level, hence 

\begin{equation*}N_{Mk}\~Binomial(N_{Sk},c_{M|S}),N_{Hk}\~Binomial(N_{Mk},c_{H|M}),\end{equation*} \begin{equation*}N_{Ik}\~Binomial(N_{Hk},c_{I|H}),N_{Dk}\~Binomial(N_{Hk},c_{D|H})\end{equation*}, 

where the first subscript indicates severity and the second indicates the population (New York, Milwaukee to May 20, Milwaukee to June 14).  

In Approach 1 (New York and Milwaukee data combined), for the unobserved level of severity (symptomatic cases) we used a prior distribution of \begin{equation*}c_{M|S}\~Beta(51.5,48.5)\end{equation*}  to represent uncertainty between 42% and 58% ; this distribution has 90% of its mass in this range, with a mean of 0.515.  The main analysis of this first approach was performed using prior information to inform the detection probabilities.  An additional “naïve” analysis was performed, in which the detection probabilities \begin{equation*}d\end{equation*}  were set equal to 1 at all levels of severity. Our prior distributions for the number of symptomatic cases in New York (overall and by age) were taken as ranging uniformly between zero and the proportion reporting ILI in the telephone survey (with the upper bound of that distribution having a prior reflecting the confidence bounds of the survey results, details in the Appendix). For Milwaukee, the prior on symptomatic cases was taken as uniform between 0 and 25% of the population. 

In Approach 2 (New York case data and telephone survey data), we made the assumption that self-reported ILI cases represented symptomatic pH1N1 infection, and used the mean and 95% confidence intervals from that survey to define a prior distribution on the number of symptomatic cases overall and by age group.  We then used observed hospitalizations, ICU/ventilator use, and fatalities along with prior distributions on detection probabilities as above to inform estimates of true numbers of hospitalizations, ICU/ventilator use, and fatalities, and these in turn were used to estimate sCHR, sCIR, and sCFR. 

The evidence was synthesized through a full probability model in a Bayesian framework, implemented in the OpenBUGS software [20] , which uses Markov chain Monte Carlo to sample from the posterior distribution.

**Table:** 

**Detection probability**	**Components**	**Distributions**	**Rationale**
\begin{equation*}d_M\end{equation*} Medically attended illness	\begin{equation*}d_{M1}\end{equation*} probability of testing, followup and reporting among medically attended cases	Uniform(.2,.35)	Data from CDC epi-aids in Delaware and Chicago [19]
\begin{equation*}d_M=d_{M1}d_{M2}\end{equation*}	\begin{equation*}d_{M2}\end{equation*} PCR test sensitivity	Uniform(.95,1)	Assumption [19]
\begin{equation*}d_{HW}\end{equation*} Hospitalization (Milwaukee)	\begin{equation*}d_{HW1}\end{equation*} probability of testing, followup and reporting among hospitalized cases	Uniform(.2,.4)	Assumption [19]
\begin{equation*}d_{HW}=d_{HW1}d_{HW2}\end{equation*}	\begin{equation*}d_{HW2}\end{equation*} PCR test sensitivity	Uniform(.95,1)	Assumption [19]
\begin{equation*}d_{IW}\end{equation*} ICU admission (Milwaukee)	\begin{equation*}d_{IW1}\end{equation*} probability of testing, followup and reporting among hospitalized cases	Uniform(.2,.4)	Assumption [19]
\begin{equation*}d_{IW}=d_{IW1}d_{IW2}\end{equation*}	\begin{equation*}d_{IW2}\end{equation*} PCR test sensitivity	Uniform(.95,1)	Assumption [19]
\begin{equation*}d_{DW}\end{equation*} Deaths (Milwaukee)	PCR test sensitivity and other detection	Beta(45,5)	Assumption [19] (mean 0.9, standard deviation 0.05)
\begin{equation*}d_{HN}\end{equation*} Hospitalization (New York City)	\begin{equation*}d_{HN1}\end{equation*} probability of performing PCR (rapid A positive or ICU/ventilated)	.27+.73 (Uniform(.2,.71))	27% of cases were ICU-admitted so received PCR test; remainder were tested if rapid A positive, which has a sensitivity of .2 [17] to .71 (sensitivity among ICU patients in NYC)
\begin{equation*}d_{HN}=d_{HN1}d_{HN2}\end{equation*}	\begin{equation*}d_{HN2}\end{equation*} PCR test sensitivity	Uniform(.95,1)	Assumption [19]
\begin{equation*}d_{IN}\end{equation*} ICU/ventilation (New York City)	PCR test sensitivity	Uniform(.95,1)	Assumption [19]
\begin{equation*}d_{DN}\end{equation*} Deaths (New York City)	PCR test sensitivity and other detection	Beta(45,5)	Assumption [19](mean 0.9, standard deviation 0.05)

**Table 1: Detection Probabilities**

## RESULTS

Table 2 shows the numbers of medically attended cases, hospitalizations, ICU visits and deaths in the two cities, with the Milwaukee data separated into the period (to May 20) for which we believe medically attended cases were consistently detected, and the period (to June 14) for which we consider only hospitalized cases, ICU admissions and deaths.

**Table:** 

	** Severity**	** **	** **	** **	** **
	Medically attended	Hospitalized		ICU-admitted	Dead
** Milwaukee**		to May 20 / to June 14		to June 14	to June 14
0-4	126 (16%)	7 (28%)	27 (18%)	5 (20%)	0
5-17	470 (60%)	6 (24%)	29 (20%)	7 (26%)	2 (50%)
18-64	189 (24%)	12 (48%)	87 (59%)	14 (52%)	2 (50%)
65+	3 (0.4%)	0	4 (3%)	1 (4%)	0
Total	788	25	147	25	4
** **					
** New York **					Dead (total) / Dead but not hospitalized
0-4		225 (23%)		44 (17%)	2 (4%) / 2
5-17		197 (20%)		51 (20%)	2 (4%) / 1
18-64		518 (52%)		147 (57%)	46 (87%) / 6
65+		56 (6%)		15 (6%)	3 (6%) / 0
Total		996		257	53 / 9

### **Table 2:** Cases at each level of severity

*Approach 1: *We considered two alternatives to estimate the ratios of interest from the combined New York and Milwaukee data, using self-reported rates of seeking medical attention to establish the denominator.  First, we obtained a naive estimate of the ratios of deaths to hospitalizations, ignoring differences in detection across levels of severity; and second, we obtained an estimate that incorporated evidence and expert opinion on the detection probabilities at each level of severity.   

The naïve estimate would suggest a median (95% credible interval (CI)) ratio of deaths to hospitalizations ( \begin{equation*}c_{D|H}\end{equation*} ) of 4.3% (95% CI 3.2%-5.5%), of ICU admissions to hospitalizations ( \begin{equation*}c_{I|H}\end{equation*} ) of 25% (95% CI 22%-27%), and of hospitalizations to medically attended cases ( \begin{equation*}c_{H|M}\end{equation*} ) of 3.1% (95% CI 2.0%-4.4%). The ratio of deaths outside of hospitals to medically attended cases ( \begin{equation*}c_{D|M}\end{equation*} ) is estimated to be 0.03% (95% CI 0.01%-0.06%).  Incorporating the prior evidence that 42 to 58% of symptomatic ILI is medically attended, this would imply a naïve estimate of the symptomatic case-fatality ratio of 0.081% (95% CI 0.049%-0.131%), a corresponding estimate of the symptomatic case-ICU admission ratio of 0.38% (95% CI 0.24%-0.58%), and an estimate of the symptomatic case-hospitalization ratio of 1.55% (95% CI 0.98%-2.32%).  If one assumes that detection probabilities are no worse at higher levels of severity than at lower levels, then these figures would be reasonable *upper bounds* on the symptomatic case-fatality and case-ICU admission ratios. 

Incorporating prior evidence of the detection probabilities at each level of severity, and thus accommodating structural and statistical uncertainties in these probabilities, we estimated that ratio of deaths to hospitalizations ( \begin{equation*}c_{D|H}\end{equation*} ) of 2.7% (95% CI 1.8%-3.8%) of ICU admissions to hospitalizations ( \begin{equation*}c_{I|H}\end{equation*}) of 17% (95% CI 12%-21%) and of hospitalizations to medically attended cases (\begin{equation*}c_{H|M}\end{equation*} ) of 2.9% (95% CI 1.6%-5.0%). The ratio of deaths outside of hospitals to medically attended cases ( \begin{equation*}c_{D|M}\end{equation*} ) is estimated to be 0.02% (95% CI 0.01%-0.04%). 

Table 3 shows the estimates for the quantities of primary interest, overall and by age group, in the analysis that incorporated prior evidence of detection probabilities.  Here, the posterior median estimate for the symptomatic case-fatality ratio is 0.048% (95% credible interval 0.026%-0.096%) and for the symptomatic case-ICU admissions ratio is 0.239% (95% CI 0.134%-0.458%).  The symptomatic case-hospitalization ratio is estimated as 1.44% (95% CI 0.83%-2.64%).  

**Table:** 

		**sCFR**			**sCIR**			**sCHR**		
age	0-4	0.026%	(0.006%	-0.092%)	0.321%	(0.133%	-0.776%)	2.45%	(1.10%	- 5.56%)
	5-17	0.010%	(0.003%	-0.031%)	0.106%	(0.043%	-0.244%)	0.61%	(0.27%	- 1.34%)
	18-64	0.159%	(0.066%	-0.333%)	0.542%	(0.230%	-1.090%)	3.00%	(1.35%	- 5.92%)
	65+	0.090%	(0.008%	-1.471%)	0.327%	(0.035%	-4.711%)	1.84%	(0.21%	- 25.38%)
	**Total**	**0.048%**	**(0.026%**	**-0.096%)**	**0.239%**	**(0.134%**	**-0.458%)**	**1.44%**	**(0.83%**	**- 2.64%)**

**Table 3: **Posterior median (95% credible interval) estimates of the symptomatic case-fatality, case-ICU and case-hospitalization ratios, by age group, based on a combination of data from New York City and Milwaukee, and survey data on the frequency of medical attendance for symptomatic cases.  

Estimates of each of these severity measures vary dramatically by age group, with the lowest severity by each measure in the 5-17 year age group. Comparing the two groups for which we have the most data, the relative risk of death for a symptomatic 18-64-year-old compared to a symptomatic 5-17 year old is 15 (95% CI 5-57). The corresponding relative risks of ICU admission and hospitalization are 5 (95% CI 2-13) and 5 (95% CI 2-12) respectively. The Bayesian framework provides a natural way to estimate confidence (measured as the posterior probability) that one rate is higher than another. The probability that severity is higher in the 18-64 age group than in the 5-17 age group is >99.9%, for each of fatality, ICU admission, and hospitalization respectively. The data are too sparse to say with confidence whether adults over 65 or under 65 have greater severity. For example, among the four age groups, the symptomatic case-fatality ratio is highest in the 18-64 year old age group with posterior probability 62.%, and in those 65 and over with probability 38%. The symptomatic case-ICU admission ratio is highest in 18-64 year olds with posterior probability 51% and in those over 65 with posterior probability 38%. The symptomatic case-hospitalization ratio is highest in 18-64 year olds with posterior probability 37% and in those over 65 with posterior probability 37%. 

*Approach 2:*  Table 4 shows the estimates for the symptomatic case-fatality, case-ICU admission and case-hospitalization ratios, by age group, when self-reported influenza-like illness is used as the denominator for total symptomatic cases.  Overall these are, respectively, sCFR=0.007% (95% CI 0.005%-0.009%), sCIR=0.028%  (95%CI 0.022%-0.035%) and sCHR=0.16% (95% CI 0.12%-0.26%). Compared to Approach 1, these estimates are nearly an order of magnitude smaller, and the age distribution differs. The relative risks for each severity in the 18-64 year old group compared to the 5-17 year old group are 7 (95% CI 3-25) for fatalities, 1.5 (95% CI 0.9-2.5) for ICU admissions and 1.4 (95% CI 0.9-2.1) for hospitalizations. The case-fatality ratio is highest in the 18-64 group with posterior probability 52%. In contrast to Approach 1, the case-ICU admission ratio is highest among 0-4 year olds, with posterior probability 79%, and the case-hospitalization ratio is highest among 0-4 year olds with posterior probability 99%. 

**Table:** 

		**sCFR**	**sCIR**	**sCHR**
age	0-4	0.004% (0.001%-0.011%)	0.045% (0.026%-0.078%)	0.33% (0.21%- 0.63%)
	5-17	0.002% (0.000%-0.004%)	0.019% (0.013%-0.027%)	0.11% (0.08%- 0.18%)
	18-64	0.010% (0.007%-0.016%)	0.029% (0.021%-0.040%)	0.15% (0.11%- 0.25%)
	65+	0.010% (0.003%-0.025%)	0.030% (0.016%-0.055%)	0.16% (0.10%- 0.30%)
	**Total**	**0.007% (0.005%-0.009%)**	**0.028% (0.022%-0.035%)**	**0.16% (0.12%- 0.26%)**

**Table 4:** Posterior median (95% credible interval) estimates of the symptomatic case-fatality, case-ICU and case-hospitalization ratios, by age group, using self-reported influenza-like illness as the denominator of symptomatic cases.

## DISCUSSION

We have estimated, using data from two cities on tiered levels of severity and self-reported rates of seeking medical attention, that approximately 1.44% of symptomatic 2009 pH1N1 patients during the spring in the United States were hospitalized; 0.239% required intensive care or mechanical ventilation; and 0.048% died.  Within the assumptions made in our model, these estimates are uncertain up to a factor of about 2 in either direction, as reflected in the 95% credible intervals associated with the estimates.  These estimates take into account differences in detection and reporting of cases at different levels of severity, which we believe, based on some evidence, to be more complete at higher levels of severity.  Without such corrections for detection and reporting, estimates are approximately two-fold higher for each level of severity.  Using a second approach, which uses self-reported rates of influenza-like illness in New York City to estimate symptomatic infections, we have estimated rates approximately an order of magnitude lower, with a symptomatic case-hospitalization ratio (sCHR) of 0.16%, a symptomatic case-intensive care ratio of 0.028%, and a symptomatic case-fatality ratio of 0.007%.  In both approaches, the sCFR was highest in adults and lowest in school-age children (5-17); data on children 0-4 and adults 65 and older were relatively sparse, making statements about their ordering more difficult.  Nonetheless, given the large number of cases in nonelderly adults, this represents a substantial shift in the burden of hospitalization and mortality from those over 65, for whom seasonal influenza is most severe [21] , to middle-aged adults, consistent with findings from previous pandemics [22] .  

These estimates are valuable for attempting to project, in approximate terms, the possible severity of a fall-winter wave of pH1N1, under the assumption that the virus does not change its characteristics.  From the 1957 and 1968 pandemics, it appears that perhaps 40-60% of the population was serologically infected, and that of those, 40-60% were symptomatic [2] [23] [24] [25] .  Current estimates of the transmission of 2009 pH1N1 range between about 1.4 and about 2.2, consistent with estimates of the reproduction numbers from prior[26] [27] [28] [29] [30] .  To convert our estimates into population impacts, one needs to make an assumption about the attack rate and its age distribution.  For each 10% of the US population symptomatically infected (with the same age distribution observed in the spring wave), our Approach 1 estimates suggest that approximately 7800-29,000 deaths (3-10 per 100,000 population), 40,000-140,000 intensive care admissions (13-46 per 100,000 population), and 250,000-790,000 hospitalizations (170-630 per 100,000 population).  These estimates will scale up and down in proportion to the attack rate; for example, they should be doubled if 20% of the population were symptomatic, producing for example 15,000-58,000 deaths, or 6-20 per 100,000 population.  Approach 2 suggests much smaller figures (for each 10% of the population symptomatic) of 1500-2700 deaths (0.5-0.9 per 100,000); 6600-11,000 ICU admissions/uses of mechanical ventilation (22-35 per 100,000); and 36,000-78,000 hospitalizations (12-26 per 100,000).  Again, these numbers should be scaled in proportion to the attack rate.

 To date, symptomatic attack rates seem to be far lower than 25% in both the completed Southern Hemisphere winter epidemic and the autumn epidemic in progress in the United States; severe outcomes seem to be considerably less numerous than those described for Approach 1 with a 25% attack rate. In New Zealand, just under 2% of the population consulted a general practitioner for influenza-like illness during the winter wave of the pandemic there (http://www.moh.govt.nz/moh.nsf/indexmh/influenza-a-h1n1-update-138-180809), consistent with an attack rate significantly lower than 25%, though somewhat higher than the GP consultation rate observed in severe seasonal flu outbreaks such as those in 2003 and 2004 (http://www.surv.esr.cri.nz/PDF_surveillance/Virology/FluWeekRpt/2004/FluWeekRpt200444.pdf).  

Our estimates reflect a level of antiviral treatment and health care capacity that will not be available in all populations.  Oseltamivir use was common in Milwaukee (Milwaukee Department of Health, unpublished data), and although the health system was stressed in both cities studied, there was no shortage of intensive care or other life-saving medical resources.  In a situation of greater stress on the health system, as has been observed in certain locations in the Southern Hemisphere [9] [10] http://www.capegateway.gov.za/eng/your_gov/3576/news/2009/aug/185589, , or in areas that lack a high-quality health system, severity might increase in proportion as adequate medical attention is less available.  Worryingly, our estimates of the proportion of symptomatic cases requiring mechanical ventilation or ICU care was approximately 4-5x our estimate of the sCFR. It is possible that a substantial proportion of those admitted to ICUs would have died without intensive care.  In populations without widespread access to intensive care, our results suggest that the same burden of disease would lead to a death rate 4-5 times higher.  Likewise, a change in the virus to become more virulent or resistant to existing antiviral drugs, or the emergence of more frequent bacterial coinfections could increase the severity of infection compared to that observed so far.

Estimates of severity for an infection such as influenza are fraught with uncertainties [1]. Our analysis has accounted for many of these uncertainties, including imperfect detection and reporting of cases, bias due to delays between events (such as the delay from illness onset to death), and the statistical uncertainties associated with limited numbers of cases, hospitalizations and deaths.  Another major source of difficulty is the spatial and temporal variation in reporting effort for mild and severe cases; for example, most jurisdictions in the United States stopped reporting mild cases on or before the second week of May, but this change varied by jurisdiction.  We have attempted to avoid this difficulty by focusing on individual jurisdictions – New York and Milwaukee – for which the approach to reporting was relatively stable over time.  One limitation is that Milwaukee changed its guidance during our surveillance period from testing of all symptomatic cases to testing of all symptomatic health care workers but only moderate to severe cases in non-health care workers.  We believe that testing policies did not change dramatically during this period, because the proportion of hospitalized case remained fairly constant; however, the sample size prior to this change in guidance was small.  Thus, our estimates should be seen as being the risk of severe outcome among persons with symptoms, possibly biased somewhat toward those with more severe symptoms.

Despite our efforts to account for sources of uncertainty, several others remain and have not been accounted for in our analysis.  First, we have assumed that for each level of severity (from medically attended up to fatal), case reporting was equal across age groups; for example, we assumed that medically attended cases were as likely to be reported for young children as for adults.  It is possible that this is not the case, for example that mild cases were more likely to come to medical attention if they occurred in children than if they occurred in adults.  If this were true, our conclusion that severity was higher in adults than children could be partly a result of differential reporting.  

 Second, the overall estimates of severity (not stratified by age group) reflect the age composition of cases in the sample we studied, especially the age composition of the lowest level of severity examined, medically attended illness.  Among medically attended cases in Milwaukee, 60% were in the 5-17 age group, the one in which severe outcomes were the least likely.  A preponderance of cases within this age group may be typical of the early part of influenza epidemics, and while it has been argued that there is a shift from younger to older age groups in seasonal influenza [31] as the epidemic progresses, there is evidence from at least the 1957 pandemic that attack rates remain higher in children than adults throughout the course of the epidemic [2] .  Since severity appears to be considerably higher in adults, then a shift in the burden of disease from children to adults as the epidemic progresses would lead to an increase in average severity.   

We note that the association between age and severity may also affect observed trends in the characteristics of cases.  The World Health Organization has noted worldwide a shift from younger to older mean age among confirmed cases (http://www.who.int/csr/disease/swineflu/notes/h1n1_situation_20090724/en/index.html).  If severity is lowest among children, this upward shift in age distribution may partially reflect a shift toward detection of more severe cases, rather than a true shift in the ages of those becoming infected.

Third, the symptomatic case-fatality, case-ICU admission and case-hospitalization ratios are dependent upon our estimates of the true number of symptomatic cases, \begin{equation*}N_{iSk}\end{equation*} and hence are sensitive to the choice of prior for these, as well as to our prior assumptions on the detection probabilities.  In particular, if the probability that symptomatic cases seek medical attention and are confirmed is lower than we assume in our prior distributions, then there are more cases than are inferred by our model, and severity is correspondingly lower than our estimates.  If the probability of detecting severe outcomes (hospitalizations, deaths, ICU) is lower than our priors reflect, then there are more severe outcomes than our model infers, so severity is correspondingly higher.  

Finally, the small sample sizes in some age groups, the over-65 year olds in particular, lead to large uncertainty about the age-specific estimates.  This level of uncertainty is reflected in the wide 95% credible intervals for the estimates.

Our two approaches yield estimates that differ by almost an order of magnitude in the severity of the infection, on each of the three measures considered.  How should planners evaluate these contrasting estimates?  The lower estimates, using the denominator of self-reported influenza-like illness in New York City, may reasonably be considered lower bounds on the true ratios.  Influenza-like illness is thought to be relatively rare in May-June, hence true influenza-like illness was probably largely attributable to pH1N1 during this period in New York.  However, self-reported ILI is notoriously prone to various biases, most of which suggest that true rates are probably lower than self-reported rates.  A previous telephone survey conducted in New York City found that 18.5% of New Yorkers reported influenza-like illness in the 30 days prior to being surveyed in late March, 2003 [32] , which represented a period of above-baseline but declining influenza activity nationally and no known influenza outbreaks in New York City [32] .  The survey was repeated in October-November, 2003, prior to the appearance of significant influenza activity, and 20.8% reported influenza-like illness in the 30 days prior [32] .  If these surveys represent a baseline level of self-reported ILI in the absence of significant influenza activity, then the approximately 12% self-reported ILI in the telephone survey is substantially lower than this out-of-season baseline, suggesting that it likely overstates the total burden if symptomatic pH1N1 disease.  The lower estimates are also broadly consistent with estimates from New Zealand, which has experienced a nearly complete influenza season [8], and from Australia (http://www.health.gov.au/internet/main/publishing.nsf/Content/cda-surveil-ozflu-flucurr.htm/$FILE/ozflu-no14-2009.pdf).  The higher estimates, on the other hand, were obtained using ratios of hospitalizations to confirmed medically attended cases and self-reported rates of seeking medical attention for ILI, which have been consistently measured in the range of about 40-60%.  It is possible that the special efforts of the New York City health department to identify pH1N1-related fatalities (including those not hospitalized) provides a fuller picture of the total number of deaths from this infection.  Interestingly, New York City reports about the same number of hospitalizations for our study period (996) as New Zealand reports up to mid-August (972), but 3.5x as many deaths (53 vs. 16) [8] .  If this discrepancy reflects more complete ascertainment of deaths in New York City, it may account for much of the difference between our higher estimates of case-fatality ratios and those from New Zealand.  Given the number of uncertainties cataloged above (which apply also to other jurisdictions within and outside the USA), we believe that our two approaches probably bracket the reasonable range of severity for the US spring wave.

Age-specific severity patterns as estimated here are largely consistent with those one would obtain by simply comparing the incidence of confirmed cases, hospitalizations, and deaths in the United States as a whole for a similar period [19] , although the estimates for persons over 65 are highly uncertain, with 95% credible intervals spanning several orders of magnitude, due to the very small number of individuals in our sample from that age group.  

The estimates provided here may be compared to those for seasonal influenza.   Compared to seasonal influenza, these estimates (assuming a 25% symptomatic attack rate) suggest a number of deaths in the United States that could range from about half the number estimated for an average year to nearly twice the number estimated for an average year [33] (Approach 1), or a range about 10-fold lower than that (Approach 2); however, the deaths would be expected to occur in younger age groups, compared to the preponderance of deaths in persons over 65 in seasonal influenza.  Such a shift in age distribution is typical for pandemics and the years that follow them [22] .  Under Approach 1, and assuming a typical pandemic symptomatic attack rate of 25%, the estimated number of hospitalizations for an autumn-winter pandemic wave is considerably more than the approximately 300,000 estimated for typical seasonal influenza [34] , whereas Approach 2 suggests a number between 1/3 and 2/3 of that observed in typical seasonal influenza.  It should be noted that most hospitalizations, and about 90% of deaths attributed to seasonal influenza are categorized as respiratory and circulatory, not including the more specific diagnoses of pneumonia and influenza; that is, they are due to myocardial infarction, stroke, and other proximate causes, but are nonetheless likely caused initially by influenza infection [35].  The deaths included in this may have reflected more directly influenza-related causes and may not reflect these indirect causes of influenza-related death.  Indeed, it is unclear whether the proportion of indirect respiratory and circulatory causes of death and hospitalization will be so high in this pandemic year, given the younger ages involved in most severe cases.  Given these differences between the estimates here based on virologically confirmed deaths and the ecological statistical approach to estimating influenza-attributable deaths and hospitalizations for seasonal influenza, it will be difficult to interpret comparisons between the two types of estimates until (after the pandemic has passed) comparisons can be made between the ecological and the confirmed-case approach to estimating burden of hospitalization and deaths.  

Our estimate of the symptomatic case-fatality ratio is lower than those provided by Garske et al. [16], which ranges from 0.11% to 1.47% overall, and between 0.59% and 0.78% in the US, but which was based on confirmed plus probable (rather than symptomatic) cases.  Garske et al. do not account for differences in reporting by level of severity; when we ignore such differences in our “naïve” analysis, we get approximately a 2-fold increase in the estimated sCFR.  This suggests that the differences in detection of mild and severe cases may have been greater in the data sets used by Garske et al. than in those we have examined.  Nishiura et al [36] estimate that between 0.16% and 4.48% of confirmed cases in the United States and Mexico were fatal.  Wilson and Baker [37] , on the other hand, use a denominator of infections (rather than symptomatic or confirmed cases) and estimate a range of CFR from 0.0004% up to 0.6%. Our estimates fall in the middle part of this range. More recently, Baker et al. [8] used their estimates of the total incidence of symptomatic disease in New Zealand to estimate a sCFR of 0.005%, equal to the lower end of the credible interval for our Approach 2 estimate, and considerably below our Approach 1 estimate.   The generally downward trend in the estimates of severity reflects early ascertainment of more severe cases (e.g., mainly hospitalized cases in the early Mexican outbreak); the issue of ascertainment and its potential biasing effect on severity estimates has been discussed by each of these earlier reports.

While we have been careful to highlight uncertainties in the estimates of severity, our results are sufficiently well-resolved to have important implications for ongoing pH1N1 pandemic planning.  The estimated severity indicates that a reasonable expectation for the autumn pandemic wave in the United States is a death toll less than or equal to that which is typical for seasonal influenza, though possibly with considerably more deaths in younger persons.  If attack rates in the autumn match those of prior pandemics and hospitalization rates are comparable to our estimates using Approach 1, the surge of ill individuals and subsequent burden on hospitals and intensive care units could be large.  However, using Approach 2, estimates of hospitalizations and intensive care admissions are considerably lower.  Either set of estimates places the epidemic within the lowest category of severity considered in pandemic planning conducted prior to the appearance of pH1N1 in the United States, which considered case-fatality ratios up to 0.1% (http://www.flu.gov/professional/community/community_mitigation.pdf).  

Continued close monitoring of severity of pandemic H1N1 disease is needed to assess how patterns of hospitalization, intensive care utilization, and fatality are varying in space and time, and across age groups.  Increases in severity might reflect changes in the host population – for example, infection of persons with conditions that predispose them to severe outcomes, or increased severity might reflect changes in the age distribution of cases, for example a shift toward adults, in whom infection is more severe.  Changes in severity might also reflect changes in the virus or variation in the access and quality of care available to infected persons.  

## FUNDING INFORMATION

 AP and DDeA were funded by the UK Medical Research Council. DDeA was funded also by the UK Health Protection Agency. ML and SR were supported by Cooperative Agreements 1U54GM088558 and 5U01GM076497 of the Models of Infectious Disease Agent Study program of the US National Institutes of Health (US NIH). SR also received funding from grant 3R01TW008246-01S1 from the US NIH from the RAPIDD program of the Fogarty International Center of the US NIH and the Science and Technology Directorate of the US Department of Homeland Security. The funders had no role in study design, data collection and analysis, decision to publish, or preparation of the manuscript.

## CONFLICT OF INTEREST

ML has received consulting fees from the Avian/Pandemic Flu Registry (Outcome Sciences), sponsored in part by Roche.  All other authors declare no conflict of interest.  

## ACKNOWLEDGMENTS

We thank Michael G. Baker for helpful discussions and Carolyn Bridges for useful comments on earlier drafts.
